# Efficacy and Safety of Amorolfine Lotion 0.25% w/v Compared to Amorolfine Cream 0.25% w/w in Patients With Superficial Fungal Infections of the Skin: A Multi-center, Randomized, Open-Label, Active-Controlled, Non-inferiority Phase III Clinical Trial

**DOI:** 10.7759/cureus.60162

**Published:** 2024-05-12

**Authors:** M Kanaka Prasad Rao, Lokesh Siddananjappa, Neetu Sidana, Ashish Deshmukh, Binayak Diwari, Indrasish Podder, Surendra Kumar, Ravindra Mittal, Pavankumar Daultani, Ashok Jaiswal, Monika Chinda

**Affiliations:** 1 General Medicine, Rajiv Gandhi Institute of Medical Sciences, Srikakulam, IND; 2 Dermatology, Glow Skin Centre, Nagpur, IND; 3 Dermatology, Apex Hospitals Pvt. Ltd., Jaipur, IND; 4 Dermatology, Mahatma Gandhi Mission Medical College, Aurangabad, IND; 5 Skin and Venereal Diseases, Hi-Tech Medical College and Hospital, Bhubaneswar, IND; 6 Dermatology and Veneriology, College of Medicine and Sagore Dutta Hospital, Kolkata, IND; 7 Skin, Venereal Diseases and Leprosy, Sawai Man Singh (SMS) Medical College and Hospital, Jaipur, IND; 8 New Product Development, Zydus Healthcare Ltd., Ahmedabad, IND; 9 Medical Affairs, Zydus Lifesciences, Mumbai, IND; 10 Dermatology, Zydus Lifesciences, Mumbai, IND

**Keywords:** topical antifungals, tinea corporis, tinea cruris, superficial skin fungal infection, amorolfine

## Abstract

Background: Dermatophytosis, a major cause of superficial fungal infections, requires topical and systemic antifungals. Amorolfine, a morpholine derivative, is a new topical antifungal available in cream and lotion formulations.

Objective: To evaluate the efficacy and safety of amorolfine lotion 0.25% compared to amorolfine cream 0.25% in patients with dermatophytosis.

Methods: A multi-center randomized, two-arm, active-controlled, parallel, non-inferiority phase III clinical trial involving 284 dermatophytosis patients was conducted, with the test arm using amorolfine lotion and the reference arm using amorolfine cream. The study drugs were applied once daily in the evening for four weeks and patients were followed up for another two weeks. The primary endpoint was clinical cure, while secondary endpoints included mycological cure, composite cure, global efficacy assessment, and post-treatment relapse. Safety and tolerability were assessed.

Results: Amongst the enrolled patients, 69.9% and 68.1% of patients had tinea corporis, while 30.1% and 31.9% had tinea cruris. The majority of patients in both groups (99.3% test and 97% reference) achieved a clinical cure at the end of treatment. Mycological cure was achieved by 98.6% and 96.3% respectively. A composite cure was achieved by 98.6% in the test arm versus 96.3% in the reference arm. A total of two AEs were reported in two (1.4%) patients in the test group and three AEs were reported in three (2.1%) patients in the reference group, all of the AEs were mild and resolved within three days without supportive medication. No severe adverse effects were reported in any of the study subjects.

Conclusion: Amorolfine lotion 0.25% w/v showed a non-inferior clinical, mycological, and composite cure in dermatophytosis patients, was well-tolerated, and had a similar safety profile to amorolfine cream 0.25% w/w.

## Introduction

Infections with dermatophytes have increased in India during the last several years [1]. The disease's appearance, severity, responsiveness to therapy, and recurrence rate have all changed in tandem with its rising prevalence [2]. According to the literature, the disease's evolving pattern and its reaction to current treatments may be caused by the shift to *Trichophyton mentagrophytes* as the primary pathogenic organism or perhaps by terbinafine resistance [3]. Poor socioeconomic status can, however, have a distinct influence on adherence to treatment and other variables, such as the inappropriate use of topical corticosteroids [4].

The treatment strategy is determined by practicing professionals based on the cause, the place of infection, and the degree and severity of the lesions. Antifungal drugs used orally, topically, or in combination with one another are often used to treat dermatophytosis [5]. Since topical antifungals are so successful and have a minimal chance of systemic adverse effects, they are usually considered to be the first line of therapy for superficial, uncomplicated dermatomycoses. These drugs are produced in a range of vehicles, such as creams, lotions, gels, or sprays, depending on the area of involvement, to improve penetration and efficacy [6,7].

A special family of antifungal medications is amorolfine, a morpholine derivative. Its fungal cell membrane modification, which primarily targets ergosterol production, is the basis of its fungicidal effect. Amorolfine is a broad-spectrum antimycotic that exhibits high levels of activity (minimum inhibitory concentration < 2 mcg/ml) in vitro against a range of organisms, including dermatophytes (*Trichophyton*, *Microsporum*, *Epidermophyton*), yeasts (*Candida*, *Cryptococcus*, *Malassezia*), molds (*Hendersonula*, *Alternaria*, *Scopulariopsis*), as well as dematiaceous (*Cladosporium*, *Fonsecaea*,* Wangiella*) and dimorphic fungi (*Coccidioides*, *Histoplasma*, *Sporothrix*)[8-10]. Since 2003, the Indian Central Licencing Authority has approved topical amorolfine in two formulations: a 0.25% w/w cream for the treatment of dermatophytes-caused dermatomycosis, cutaneous candidiasis, and pityriasis versicolor, and a 5% w/v nail lacquer for the treatment of dermatophytes-caused onychomycosis, yeasts, and molds. Several clinical studies conducted in the mentioned medical conditions have consistently demonstrated the safety and efficacy of amorolfine in both topical cream and nail lacquer forms. Specifically, multiple trials have highlighted the impressive effectiveness of amorolfine 0.25% w/w cream in treating dermatophytosis patients, achieving higher rates of both clinical and mycological cure [8,11-17].

In the year 2020, M/s. Zydus Healthcare Ltd. developed a new topical formulation of amorolfine as amorolfine lotion 0.25% w/v to be used specifically in case of extensive lesions and lesions in hairy body areas. The current phase III clinical study was planned to evaluate and compare the antifungal efficacy and safety of amorolfine lotion 0.25% w/v with amorolfine cream 0.25% w/w in patients with dermatophytosis (tinea cruris and tinea corporis).

## Materials and methods

Study design

A prospective, randomized, two-arm, active-controlled, parallel, assessor-blind, multicenter, non-inferiority, phase III clinical trial was conducted at the following sites in India: MGM Medical College and Hospital, Aurangabad, Sparsh Hospitals and Critical Care Private Limited, Bhubaneswar; Sawai Man Singh (SMS) Medical College and Attached Hospitals, Jaipur; NKP Salve Institute of Medical Sciences and Research Centre and Lata Mangeshkar Hospital, Nagpur; Government Medical College and Government General Hospital, Srikakulam; College of Medicine and Sagore Dutta Hospital, Kolkata; Apex Hospitals Pvt. Ltd., Jaipur. 

Due to the difference in the physical characteristics of the study drugs, the test drug being a lotion formulation and the reference drug being a cream formulation, double-blinding was not feasible and hence, this study was planned to be assessor-blind. The clinical signs and symptoms as well as the adverse effects (AEs) were evaluated by the investigator / co-investigator who had been assigned the duty of assessor at the respective clinical trial sites. The assessor was not involved in the randomization, study treatment allocation, and accountability. The assessor was unaware of the study treatment received by the patients.

Study subjects

The study included both genders, 18-65 years of age, with acute symptomatic tinea corporis or tinea cruris limited to a single body region and with limited involvement i.e., < 5 skin lesions with a maximum diameter of < 5 cm, mycological diagnosis of tinea corporis or tinea cruris confirmed by detection of fungal hyphae on a microscopic potassium hydroxide (KOH) test, patients with a total clinical score of at least 5 (range: 0-9). These patients were willing to provide written informed consent and comply with the protocol requirements.

The study excluded individuals with hypersensitivity to amorolfine, extensive or disseminated tinea infections, other types of tinea infections, skin lesions with secondary bacterial infections, other dermatological conditions, patients with uncontrolled systemic diseases or immunosuppressive medications, hepatic or renal dysfunction, prior use of topical antifungal agents, corticosteroids, antihistamine agents, pregnant or lactating females, female patients of childbearing potential unwilling to use effective contraception, alcohol and/or drug abuse history, or participation in another clinical trial within three months prior to screening. These exclusion criteria were designed to ensure accurate diagnosis and treatment of tinea infections.

Study methodology

This pre-licensure clinical trial was approved by the Central Licencing Agency and it was registered on CTRI portal (CTRI/2020/09/027948) before the initiation of the screening of patients. The trial was also approved by the Institutional Ethics Committees of all the participating sites. The patients were screened and randomized as per a single central computer-generated randomization plan that was generated by the sponsor and selected randomization numbers were provided to each investigator. The patients were randomized in a 1:1 ratio in the test and the reference arm and were advised to apply amorolfine lotion or amorolfine cream over the affected skin areas and immediate surrounding (approx. 1 inch) healthy skin once daily in the evening for four weeks. The treatment period included a biweekly follow-up i.e. at Week 2 and Week 4, which was concluded as the end of the treatment. A follow-up for an additional two weeks was marked as the end of the study. Patients were also provided dosing cards to record the daily use of the study drug for assessment of treatment compliance.

Study endpoints

The patients were assessed for efficacy and safety. The primary endpoint was defined as a clinical cure at the end of the treatment when the patient’s total clinical score (TCS) was ≤ 2 with no itching. The secondary endpoints included mycological cure (no fungal hyphae on microscopic KOH test) at the end of the treatment, composite cure at the end of the treatment, global assessment of efficacy at the end of the treatment, and clinical and mycological relapse during the post-treatment follow-up.

Scoring of clinical signs (erythema and scaling) and symptoms (itching) was assessed on a 4-point scale ranging from grade 0 i.e. no signs and symptoms to grade 3 i.e. severe signs and symptoms. The sum of scores of these three signs and symptoms was considered as TCS. The mycological cure was assessed based on scrapings from the skin lesions for microscopic KOH to evaluate the presence of fungal hyphae. TCS and mycological scraping were evaluated during screening, follow-up visits, the end of the treatment, and the end of the study. The patients who had achieved both the clinical and mycological cures at the follow-up and end of treatment were considered patients with composite cures. At the end of post-treatment follow-up, the patients who had again presented with clinical signs or symptoms at the treated area(s) or had microscopic KOH test positive were considered to have a clinical or mycological relapse respectively. The global assessment of efficacy was assessed by the investigator at the end of the treatment on a 6-point scale (Table 1) [18,19].

**Table 1:** Global assessment of efficacy score at the end of treatment Source: Anto et al. [18] and Chandana et al. [19]

Score	Criteria
-1	Exacerbation (flare-up at the site of treatment)
0	Unchanged
1	Mild improvement (<50% clearance)
2	Moderate improvement (50% to 75% clearance)
3	Excellent improvement (75% to 100% clearance)
4	Cleared (100% clearance)

Safety was assessed by recording the AEs occurring during the entire course of the study. Hematological and biochemical laboratory investigations were carried out during screening and at the end of treatment. All abnormalities found in the physical examination (including vitals) and clinically significant abnormalities in the laboratory investigations were to be recorded as AEs. The observed or volunteered AEs regardless of a study group or suspected causal relationship to the study drug were recorded.

The investigator rated the overall tolerability of the study treatment at the end of the treatment on a 4-point scale such as excellent, good, fair, or poor (Table 2) [20]. The AEs considered related to the study drug only were considered for tolerability grading by the investigator.

**Table 2:** 4-point Tolerability scale Source: Walsh et al. [20] AE: adverse event

Tolerability grade	Criteria
Excellent	No AE reported
Good	Mild AE(s) reported which subsided with or without medication and did not necessitate stoppage of study drug
Fair	Moderate AE(s) reported which subsided with or without medication and did not necessitate stoppage of study drug
Poor	Severe or serious AE(s), or AE(s) which necessitated stoppage of study drug

Statistical analysis

The sample size was calculated based on the primary endpoint of the proportion of patients achieving clinical cure at the end of the treatment. A total of 240 patients (Test: 120, Reference: 120) were required to achieve the non-inferiority of the test drug as compared to the reference drug at 90% power and at 2.5% one-sided level of significance, assuming that at least 85% patients will achieve clinical cure at the end of the treatment [10] with no difference between the test group and the reference group, and considering the non-inferiority margin of -15% based on the literature [21]. Considering the drop-out rate of around 15%, 282 patients were planned to be enrolled in the study with a 1:1 allocation ratio (i.e. Test: 141, Reference: 141).

The categorical variables such as the proportion of patients achieving clinical, mycological, or composite cure at the end of the treatment, clinical and mycological relapse during post-treatment follow-up, and incidence of AEs were compared between the study groups using Fisher’s exact test while continuous variables such as the score of global assessment of efficacy were compared between the study groups using unpaired t-test. The test drug was considered non-inferior to the reference group if the lower limit of 95% confidence interval for the difference between the test and the reference group for the proportion of patients achieving clinical cure at the end of the treatment was above the non-inferiority margin (-15%). The non-inferiority of the test drug as compared to the reference drug for patients achieving mycological and composite cure at the end of the treatment was also evaluated using the same approach.

## Results

A total of 284 patients with dermatophytosis were evaluated for efficacy and safety. The baseline demographics and characteristics of the disease condition of the patients are mentioned in Table 3 respectively.

**Table 3:** Baseline demographics and characteristics of disease condition in the patient population #Data presented as n (%) ^Data presented as mean ± SD $Greatest surface diameter for the skin lesion * p-value<0.05 was considered as statistical significance TCS: total clinical score

Parameters		Test group (N = 143)	Reference group (N = 141)	P-value
Age (years) ^		37.5 ± 11.8	35.3 ± 11.8	0.12
Gender#	Male	81 (56.6%)	82 (58.2%)	0.81
	Female	62 (43.4%)	59 (41.8%)	
Height (cm) ^		162.2 ± 7.9	162.4 ± 7.2	0.77
Weight (kg) ^		64.8 ± 9.1	64.8 ± 7.8	0.97
Body mass index (kg/m2) ^		24.6 ± 2.4	24.5 ± 2.2	0.89
Type of tinea infection#	Tinea corporis	100 (69.9%)	96 (68.1%)	0.8
	Tinea cruris	43 (30.1%)	45 (31.9%)	
No. of skin lesions^		2.9 ± 1.1	2.8 ± 1.1	0.49
Greatest surface diameter (cm) ^$		3.4 ± 0.7	3.3 ± 0.6	0.62
Clinical score^	Erythema	2.5 ± 0.7	2.5 ± 0.6	1
	Scaling	2.3 ± 0.6	2.3 ± 0.6	0.97
	Itching	2.1 ± 0.6	2.1 ± 0.5	0.53
	TCS	6.9 ± 1.3	6.9 ± 1.2	0.78

Assessment of efficacy

The test drug was shown to be non-inferior to the reference drug for the proportion of patients achieving clinical cure, mycological cure, and composite cure at the end of treatment as shown in Table 4.

**Table 4:** Assessment of efficacy P-value by Fisher exact test; test group vs. reference group * p-value <0.05 was considered of statistical significance

Parameters	Test group (N=140) (n[%])	Reference group (N=135) (n[%])	Test-reference (95% CI)	P-value
Patients achieving clinical cure at the end of treatment (primary endpoint)	139 (99.3%)	131 (97.0%)	2.2% (-1.6%, 6.1%)	0.21
Patients achieving mycological cure at the end of treatment	138 (98.6%)	134 (96.3%)	2.3% (-2.2%, 6.7%)	0.28
Patient achieving composite cure at the end of treatment	138 (98.6%)	130 (96.3%)	2.3% (-2.2%, 6.7%)	0.28

**Figure 1:**
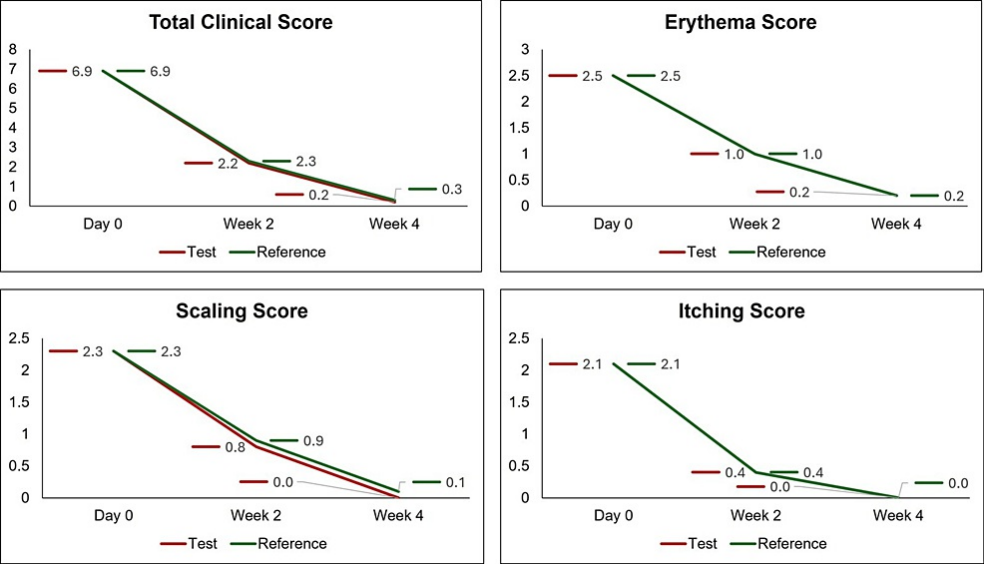
Clinical score at the baseline and during the treatment period

The mean TCS at the screening visit was 6.9 ± 1.3 and 6.9 ± 1.2 in the test group and the reference group respectively. In the test group, the mean TCS reported at the 2-week follow-up after treatment and at the end of treatment was 2.2 ± 1.8 and 0.2 ± 0.5 respectively. Likewise, in the reference group, the mean TCS reported at the two-week follow-up after treatment and at the end of treatment was 2.3 ± 1.7 and 0.3 ± 0.8, respectively. There was a statistically significant fall in TCS in both study groups at the two-week follow-up after treatment and at the end of treatment as compared to the screening visit (P<0.0001). Further, the mean TCS including the mean scores of clinical signs and symptoms were comparable between the study groups at the baseline and during the treatment period (P>0.05) (Figure 1). The mean score of global assessment of efficacy at the end of treatment was 3.8 in both the test and reference study groups and the difference between the study groups was statistically not significant (P=0.66). No patient had a clinical relapse or mycological relapse during the last two weeks of follow-up after the end of treatment in either of the groups. The efficacy results described above belong to the PP population. The results reported in the mITT population were similar to the PP population (data not shown).

Assessment of safety

A total of two AEs were reported in two (1.4%) patients in the test group and three AEs were reported in three (2.1%) patients in the reference group. The proportion of subjects for whom the AEs were reported was comparable between the study groups (P=0.68). All the AEs in both the study groups were mild (grade 1) in severity and resolved completely within three days of occurrence without any supportive medication. No serious AE (SAE) was reported in any subject.

In the test group, 100.0% of patients were given an ‘excellent’ grade, while in the reference group, 140 (99.3%) patients were given an ‘excellent’ grade and the only remaining patient (0.7%) was given a ‘good’ grade of tolerability. The overall tolerability evaluation was comparable between the study groups (P=0.50).

## Discussion

Superficial fungal illnesses known as tinea corporis and tinea cruris can affect both healthy and immunocompromised individuals. These illnesses are thought to be the most common in India, affecting people of all ages. There are major detrimental repercussions of dermatophytosis on one's social, psychological, occupational, and health. Dermatophytosis treatment serves many purposes other than appearance. A persistent illness can significantly lower one's quality of life [12].

Topical antifungal therapy is the mainstay in the management of tinea infections. The common class of drugs used are azoles, allylamines, morpholine derivatives, and pyridine derivatives [22].

Amorolfine is a unique antifungal, that acts on two different enzymes involved in sterol biosynthesis, which results in the depletion of ergosterol. This dual mechanism of action makes amorolfine a potent fungistatic and fungicidal agent [10]. According to the clinical literature available, amorolfine 0.25% cream when compared to clotrimazole 1% cream in patients with tinea corporis showed comparable results in terms of clinical and mycological cure. They also showed similar safety profiles in a treatment period of four weeks [12].

In another study, amorolfine 0.5% cream was compared to bifonazole 1% cream in patients with dermatomycosis for a treatment period of six weeks and a follow-up of three weeks. The results showed that 83.3% of patients from the amorolfine group and 78.9% of patients from the bifonazole group achieved clinical and mycological cures. There was no significant difference between the two drugs in clinical and mycological cure rates and tolerance [13].

Amorolfine used in combination with other antifungal agents like ketoconazole, terbinafine, itraconazole, and fluconazole has been seen to cause an increase in fungistatic activity against *T. mentagrophytes* [23].

As amorolfine 0.25% cream was already approved in India, the intent of developing amorolfine 0.25% lotion was to have a formulation that would be more suitable for use in hot climatic conditions, and would possibly have better penetration due to lesser viscosity. And, in case of extensive lesions, a lotion form would have better spreadability, and in the hairy areas like the groin, scalp, etc. would be preferred over the cream formulation.

Therefore, this study was done to analyze the efficacy and safety of amorolfine 0.25% lotion compared to amorolfine 0.25% cream in Indian patients suffering from dermatophytosis (tinea cruris and tinea corporis). As compared to amorolfine 0.25% cream, the amorolfine 0.25% lotion demonstrated non-inferior results for the clinical cure which was considered as the primary endpoint. Similar results were observed in the test and the reference arm when compared for the secondary endpoints of mycological cure, composite cure, and global assessment of efficacy. Furthermore, from the safety point of view, the proportion of patients for whom the AEs were reported was comparable between the study groups. There was no clinically significant abnormality reported in the hematological and biochemical laboratory investigations performed at the end of treatment in any of the patients in the study groups. In terms of overall tolerability, both arms showed comparable results.

The study had certain limitations; it was not a double-blind study, there was no other active comparator, fungal cultures were not taken, and it had a limited post-treatment follow-up period. The patients included in the study had symptomatic tinea infection in a single body region with limited involvement.

## Conclusions

The efficacy and safety of amorolfine lotion 0.25% w/v were studied in comparison to amorolfine cream 0.25% w/w in patients with dermatophytosis. All the efficacy endpoints studied for determining the clinical and mycological cure rate in patients with dermatophytosis showed that amorolfine 0.25% w/v lotion was comparable to amorolfine 0.25% w/w cream. Both the lotion and cream formulations were found to be safe and well-tolerated.
